# Automated Analysis of Time-Lapse Imaging of Nuclear Translocation by Retrospective Strategy and Its Application to STAT1 in HeLa Cells

**DOI:** 10.1371/journal.pone.0027454

**Published:** 2011-11-18

**Authors:** Fujun Han, Peizhou Liang, Feifei Wang, Lingyun Zeng, Biliang Zhang

**Affiliations:** 1 Laboratory for RNA Chemical Biology, Guangzhou Institutes of Biomedicine and Health, Chinese Academy of Sciences, Guangzhou, People's Republic of China; 2 Department of Otorhinolaryngology, People's Liberation Army No. 458 Hospital, Guangzhou, China; 3 School of Biochemistry, Medical Sciences, University Walk, Bristol, United Kingdom; 4 Department of Neuroendocrine, People's Liberation Army No. 458 Hospital, Guangzhou, China; 5 State Key Laboratory of Respiratory Diseases, Guangzhou Institute of Respiratory Diseases, Guangzhou, China; University of Connecticut, Storrs, United States of America

## Abstract

Cell-based image analysis of time-lapse imaging is mainly challenged by faint fluorescence and dim boundaries of cellular structures of interest. To resolve these bottlenecks, a novel method was developed based on “retrospective” analysis for cells undergoing minor morphological changes during time-lapse imaging. We fixed and stained the cells with a nuclear dye at the end of the experiment, and processed the time-lapse images using the binary masks obtained by segmenting the nuclear-stained image. This automated method also identifies cells that move during the time-lapse imaging, which is a factor that could influence the kinetics measured for target proteins that are present mostly in the cytoplasm. We then validated the method by measuring interferon gamma (IFNγ) induced signal transducers and activators of transcription 1 (STAT1) nuclear translocation in living HeLa cells. For the first time, automated large-scale analysis of nuclear translocation in living cells was achieved by our novel method. The responses of the cells to IFNγ exhibited a significant drift across the population, but common features of the responses led us to propose a three-stage model of STAT1 import. The simplicity and automation of this method should enable its application in a broad spectrum of time-lapse studies of nuclear-cytoplasmic translocation.

## Introduction

The movement of proteins such as transcription factors between the cytoplasm and nucleus is of great biological importance in many signaling pathways [1]. Time-lapse imaging of proteins that shuttle between nuclei and cytoplasm is also an area of increasing interest to systems biologists who are tracking protein behaviors in cells over time for modeling [2]. The most advantageous is to study the native state of cells with minimal distortions of cell morphology or function. However, most automated image analysis systems currently perform well only with fixed cells [3], [4], [5], [6]. Such experiments can also be analyzed manually, but the volume and complexity of the data generated are huge.

Some methods [7], [8], including software, such as CellTracker [6], introduce different strategies applicable for time-lapse imaging of nuclear-cytoplasmic translocation of fluorescently tagged proteins. Most of these studies focus on improving the possibilities for image analysis and hence present two major limitations. Firstly, the algorithms are often too profound for users to interpret, leading to difficulties in the applications. Secondly, image processing may be difficult under circumstances, such as incomplete nuclear-cytoplasmic translocation which causes ambiguous nuclear boundaries, or faint cellular fluorescence. Also, cells, particularly transiently transfected cells, may display fluorescence that varies significantly in intensity. These phenomena are common in live-cell imaging, but all create difficulties for differentiating nuclei from cytoplasm, even manually. Indeed, very few automated image analysis techniques can potentially satisfy the requirements imposed by live cell imaging and analysis at the individual cell level [9], [10], [11]. To the best of our knowledge, there is no system available which enables to track and identify a large volume of dynamic cellular image data of protein nuclear transport automatically and effectively.

**Figure 1:**
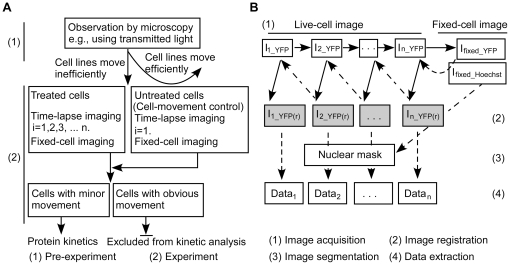
Flowchart for the time-lapse imaging system. (A) Design of the experiment. (B) Diagram of the automated image analysis. Immediately after time-lapse imaging, cells were fixed and stained with Hoechst (step 1). With I_fixed_YFP_ as the reference, I_i_YFP_ was registered and generated I_i_YFP(r)_. I_i−1_YFP(r)_ was then generated after registration with I_i_YFP(r)_, and finally, I_1_YFP(r)_ was generated (Step 2). I_fixed_hoechst_ was segmented to produce nuclear masks (Step 3), which were applied to process the registered time-lapse images (Step 4). *i = n, n−1,…, 3, 2*, represents images collected at *i*×10 min; *(r)* represents a registered image.

The first crucial step to differentiate nuclei from cytoplasm is achieved by image segmentation [12], . Convincing segmentation requires images with high contrast, which is sometimes difficult to achieve in live-cell imaging, but much easier in fixed-cell imaging. For cells that undergo little morphological change during a time-lapse experiment, it is feasible to perform “retrospective” analysis (Fig. 1A). In this analysis, cells are fixed and stained at the end of an experiment to acquire high-contrast images, which are segmented into binary masks to process the time-lapse images (Fig. 1B). Two questions then arise and the method presented here resolves both. One is how to find the same field after fixing the cells. The equipment of XY positioning stages in imaging platforms such as slide-based cytometry [15], together with techniques of image registration, enables accurate correspondence of a fixed-cell image to the previous time-lapse images. The other question is how to separate the contributions of cell movement and protein translocation within the measured fluorescence. If a cell changes morphology during the experiment this should result in mismatch between its nuclear mask at the end of the experiment and its initial nuclear position. If a target protein displays fluorescence in different patterns between the nucleus and cytoplasm, the measured value resulting from a mismatched mask should be different from a matched mask. The difference revealed by matched and mismatched masks can thus be used to identify cells that have moved. Based on these considerations, we have developed a simple and reliable method to process time-lapse images of nuclear-cytoplasmic translocation (Fig. 1).

Signal transducers and activators of transcription 1 (STAT1) belongs to a family of transcription factors downstream of many cytokines and cell growth factors, one of which is interferon gamma (IFNγ) [16]. In non-activated cells STAT1 proteins are mostly in the cytoplasm in an inactivate form [16]. Upon stimulation by IFNγ, cytoplasmic STAT1 translocates to the nucleus and activates transcription by binding to specific recognition sites on the DNA [16]. We adopted this well-characterized and biologically important signaling system to set up and validate this automated, retrospective image analysis protocol. To our knowledge, for the first, we quantified the complexity in the dynamics of STAT1 translocation within cell populations. This method offers a computational framework to assess the complexity of cellular heterogeneity in the dynamics of shuttling proteins.

## Materials and Methods

### Cell culture and transient transfection

Plasmid STAT1-YFP was generated by inserting cDNA encoding the human STAT1 protein into pEYFP-C1 (BD Biosciences Clontech, Palo. Alto, CA). HeLa cells (ATCC CCL-2) were cultured in Dulbecco's modified Eagle's medium (DMEM; Gibco, Shanghai, China) supplemented with 10% fetal bovine serum (FBS) in a humidified incubator at 37°C with 5% CO_2_. 6000 cells were plated per well in 96-well plates (Bioscience, San Diego, CA, USA) and cultured for 12 h. Cells were then transfected using Lipofectamine 2000 (Invitrogen, Shanghai, China) according to the manufacturer's instruction. Cells were maintained in phenol red-free DMEM (Gibco) with 10% of FBS for 12 h after the transfection.

### Time-lapse imaging

Plates were moved to the stage of a BD Pathway 855 bioimager (BD Bioscience) in a 5% CO_2_ atmosphere and 37°C heated chamber. Cells were treated with 100 U/ml IFNγ (Sigma, Shanghai, China) [17], [18] or an equivalent volume of the solvent (as a control for fluorescence variation in the time lapse), and images were collected every 10 min for 120 min. After collecting the 120 min image, cells were processed for fixed-cell imaging. Images of untreated cells were collected once at time 0 and then fixed as a control for cell movement.

### Fixed-cell imaging

Cells were fixed in 4% paraformaldehyde and permeabilized with 0.2% Triton X-100 in PBS, and stained with 1 µg/ml Hoechst 33342 (Sigma). The process from the end of the time-lapse imaging to the start of the fixation was completed in less than 3 min. Again, plates were moved into the Pathway and images taken.

### Image acquisition

Automated laser-focus image capturing was performed at room temperature using the BD Pathway 855 bioimager (BD Biosciences, San Jose, CA) controlled by BD Attovision™ 1.6.1 software (BD Bioscience, San Jose, CA) equipped with a camera (ORCA-AG; Hamamatsu Photonics, Hamamatsu, Japan) using an objective (40× Universal Apochromat; 0.75 numerical aperture; Olympus, Tokyo, Japan). All the images were 12-bit grayscale and of size 512×672 pixels, corresponding to a 210×160 µm field of view. The images were captured as single montage, and one image for each well was acquired. For each experiment, a total of 90 fields (50–90 cells per field) were collected. These were 90 sets of time-lapse series that contained 90×13 live-cell images and 90×1 fixed-cell images. This experiment was repeated three times independently (the average transfection efficiency was 19%), with a maximum number of cells captured being 7708 during a time course. YFP images were obtained with excitation filter (488/20) and emission filter (520/25), and Hoechst staining was visualized with excitation filter (360/10) and emission filter (435LP). The specimens were illuminated to the minimum necessary for sufficient signal-to-noise ratio for phenotyping, with exposure times of 200 ms for YFP and 100 ms for Hoechst. In all cases, exposure times and other settings were kept constant to allow equal comparisons between experiments. Upon completion of a 96-well plate, all the images were displayed in a plate view by BD AttovisionTM 1.6.1 software, and visual inspection was performed.

### Image analysis

The automated program we developed using Matlab7.6 software (The MathWorks, Natick, Mass, USA) proceeds in three major steps: image registration, segmentation and data extraction. For description, the fixed-cell images were denoted by I_fixed_YFP_ and I_fixed_Hoechst_, and the time-lapse images by I_i_YFP_, *i = 1,2,3,…,n* (representing STAT1-YFP images collected at *i*×10 min; *n = 1* for the cell-movement control).

#### 1) Image registration

Image registration was carried out by the normalized cross-correlation method (the Image Processing Toolbox of Matlab). The last image, I_n_YFP_, was registered with I_fixed_YFP_ as the reference. Because adjacent images shared more similarities, we sequentially registered I_i_YFP_ with I_i+1_YFP(r)_ as the reference, _(r)_ represents a registered image (Fig. 1B). Source codes of the algorithms presented in this section are available by the authors (File S1).

#### 2) Segmentation

I_fixed_Hoechst_ was segmented to identify the nuclei. Briefly, the nuclear edges were detected by Laplacian of Gaussian method, and a flood-fill algorithm was used to produce the masks of the nuclei [19]. This method missed some real nuclei and generated false masks due to its sensitivity to noise [20]. Otsu's threshold segmentation [21], [22] was used to reduce this effect. Connected nuclei in the masks were separated using a watershed splitting method [23], [24]. Finally, the masks covering less than half of an average nuclear area (empirically determined) were removed.

I_1_YFP_ had the strongest cytoplasmic fluorescence in the time-lapse image sequence, and thus was segmented by Otsu's method [21], [22] to obtain the foreground (areas with cells). To assign the nucleus of each cell, the foreground was processed by the seeded watershed segmentation [24] with the segmented nuclei as the seeds. Because this method is not based on actual cell boundaries, the cell boundaries identified may not be well-defined. To reduce the confusing cell boundaries, the seeded nuclei were dilated and then intersected with the cytoplasmic mask to produce a final cytoplasmic mask. The distance of the dilation is empirically determined and denoted by d; d = 6, 7, 8, … or 20.

Quality control was integrated as part of nuclear and cytoplasmic segmentation, and the aim was to eliminate segmentation errors. For this purpose, we first calculated cell parameters, including mean and standard deviation (s.d.) of Hoechst and YFP intensities, and measures of shapes such as nuclear perimeter, nuclear solidity, and cell perimeter. Deviated values indicate segmentation errors. In addition, cells that resided at the edges of any individual image during a time course were excluded from quantification.

#### 3) Evaluation of nuclear segmentation

Three descriptors were defined- match, mismatch and false mask. A matched mask is defined by a nuclear mask which falls into the corresponding nucleus and covers more than 50% of the nuclear area (as detailed in the section of **Segmentation**). This definition includes an identified nucleus smaller than the real one, because we later proved that both s.d. and mean of the measured fluorescence of the target protein varied only slightly. If some area of a nuclear mask is out of the nucleus, e.g., overlapping with the cytoplasm, the mask is considered to be a mismatch. False mask denotes a nuclear mask caused by artifact fluorescence.

#### 4) Data extraction

The pixels adjacent to the boundary of a nuclear and the cytoplasmic mask were removed by eroding the nuclear mask to reduce the cross-contamination between the nuclear and cytoplasmic fluorescence. The distance of the erosion is empirically determined and denoted by *e*; *e* = 2, 3, 4, 5, or 6. In the image analysis, parameters were defined as follows: 1) **Nuclear∶cytoplasmic ratio (N∶C ratio):** the ratio of nuclear to cytoplasmic mean fluorescence intensity. The ratio was normalized to 1 at time 0 (before stimulation). 2) **Nuclear accumulation (NA):** The mean fluorescence intensity of a nucleus divided by the mean of the same nucleus at time 0 to be normalized for different protein expression levels between cells. 3) **Nuclear increment (NI):** To acquire direct information on the speed of protein nuclear translocation, NI (***ΔI_i_***), was defined and normalized as follows. ***ΔI_i_*** = (***I_i_YFP_***_*(R)*_−***I_i−1_YFP_***_(R)_)/***I_1_YFP_***_*(R)*_, *i* = *2*,*3*,…,*n*. 4) **Difference variation:** Different parameter settings for the erosion (*e*) and dilation (*d*) were tested and only those with maximal match between the masks and the corresponding regions were used to process the images. Even though the masks were all in the range of the corresponding nuclei or the cytoplasm, the measured values, NA or N∶C ratio, were still different. Nonetheless, the more reliable descriptor should generate data with smaller variation. To evaluate the reliability, difference variation was defined and normalized as: |***Value 1***−***Value 2***|/(***Value 1***+***Value 2***), where ***Value*** donates N∶C ratio or NA, ***1*** and ***2*** represent data produced by two masks, respectively.

## Results and Discussion

Most automated image analysis methods aim to achieve better segmentation to analyze time-lapse images. As a result, these methods are bottlenecked by a wide range of biological factors, as described in the Introduction. Herein, we presented a “retrospective” method to overcome the bottlenecks. In the method, cells were fixed and stained with Hoechst at the end of the experiment and the nuclear-stained image was segmented to binary masks to process the time-lapse images. Because the nuclear-stained image exhibited high contrast and was independent of the fluorescence of a target protein, our method was not constrained by these biological conditions.

### Establishment of the method: identification of cells with morphological changes

**Figure 2:**
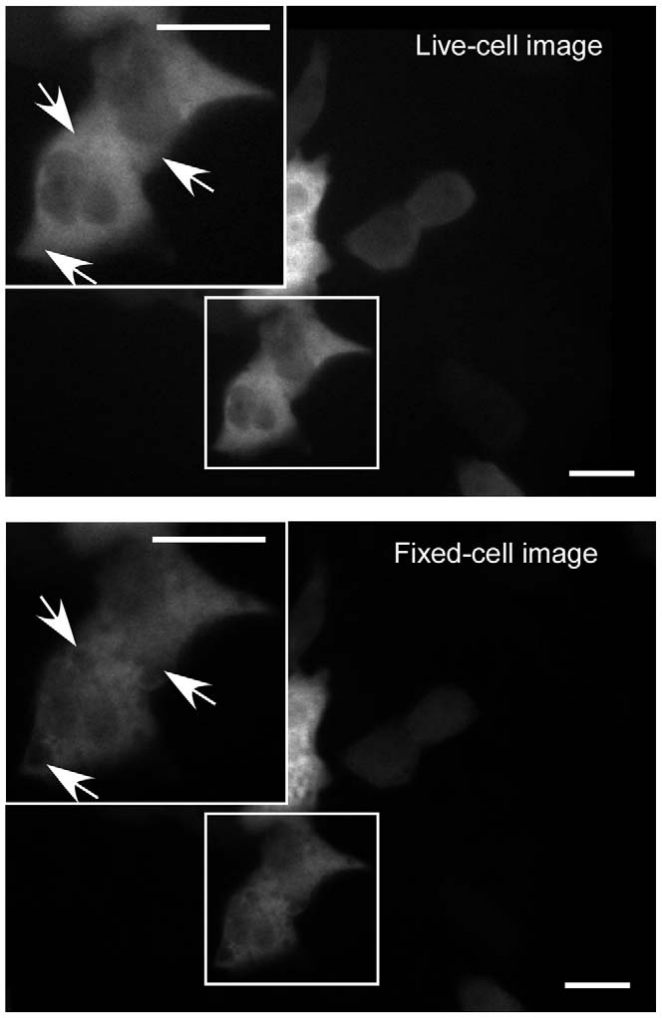
STAT1-YFP fluorescence before and after fixation. STAT1-YFP was transiently expressed in Hela cells. YFP images of the untreated cells were collected, the cells were then fixed and YFP images of the same field were taken. Here shows representatives of a registered live-cell image and the fixed-cell image. For each image, a magnified view of the field is shown in an inset. Arrows point to areas with obvious change in fluorescence intensity. Images with black borders are the registered images; the borders indicate the distance by which the original YFP image is shifted away from the fixed-cell images. Scale bar, 20 µm.

A prerequisite for this method is that no major cell movement occurs during the time-lapse period (Fig. 1A). However, cells do move over time and it is desirable to separate the contributions of cell movement and protein translocation to the measured fluorescence of a target protein. This can be achieved if the measured fluorescence between stationary cells (represented by matched nuclear masks) and migrated cells (represented by mismatched nuclear masks) are different. To define stationary cells (cell-movement control), images of untreated HeLa cells transiently expressing STAT1-YFP were taken at a single time (time 0) and the cells immediately fixed for nuclear staining. As expected, in these cells STAT1-YFP was mostly cytoplasmic with a low level of nuclear fluorescence (Fig. 2). However, we found that STAT1-YFP fluorescence was unevenly decreased after fixation (Fig. 2), thus could not be applied for this purpose. Therefore, the live-cell images best represented the fluorescence properties of the target protein in stationary cells and were used as the cell-movement control in further experiments.

**Figure 3:**
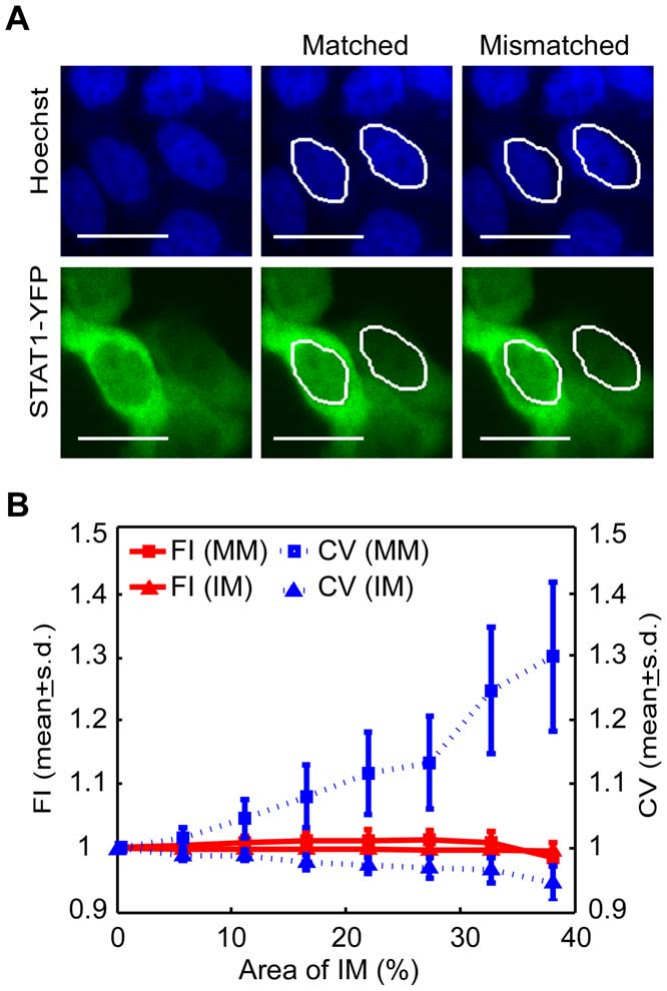
Mismatch of nuclear masks with the nuclei increased the s.d. of the measured fluorescence of the target protein. The experiment of time-lapse imaging of STAT1-YFP nuclear translocation was repeated three times independently with a cell-movement control (Materials and method) each time. The identified STAT1-YFP expressing cells from the cell-movement controls were pooled together. 300 matched nuclear masks, randomly selected from this pool, were shifted randomly out of their original positions along the horizontal, and (or) vertical, and (or) rotational directions to generate mismatched masks. The intersections between the mismatched and the original masks were used to mimic masks smaller than the real nuclei (not shown). (A) A simplified illustration of mismatched masks generated by artificially shifting a registered live-cell image of YFP emission. The original masks, shown as white outlines in the middle panel, were artificially shifted 8 pixels in left-to-right direction, generating mismatched masks (right panel). (B) For the generated mismatched masks (MM) and intersected masks (IM), the coefficients of variance (CV) (ratio of s.d. to mean) and mean of YFP fluorescence intensity were plotted against the area of the IM (represented by area ratio of the IM to the original mask), separately. Fluorescence intensity, FI. The CV and mean before the artificial variation was normalized to 1. The CV is represented by dashed lines and the scale on the right axis. The mean is represented by solid lines and the scale on the left axis. Scale bar, 20 µm. This experiment was repeated three times independently, and similar results were obtained. Here shows a representative experiment.

Next, fluorescence differences displayed by stationary and migrated cells were analyzed. As a first step to validate that this method, based on fluorescence intensity, can be applied to cells, mismatched masks were generated artificially and tested. 300 matched nuclear masks were randomly selected from the cell-movement control. These masks were shifted randomly out of their original positions along the horizontal, and (or) vertical, and (or) rotational directions (Fig. 3A). The intersections between the mismatched and original masks were used to mimic nuclear masks smaller than the real nuclei. The mismatch resulted in more obvious increase of the s.d. (dispersion; blue squares in Figure 3B) than the mean (central tendency; red squares in Figure 3B) as STAT1-positive cytoplasm became included in the mask. In contrast, in the analysis of the intersected masks, both parameters changed in much less degree (triangles, Fig. 3B). We considered that the s.d. and mean could provide a way to differentiate matched or mismatched nuclear masks. If a nuclear mask is smaller than an actual nucleus, the mean fluorescence intensity changed only slightly (red triangles, Fig. 3B), hence the value can represent true protein translocation.

**Figure 4:**
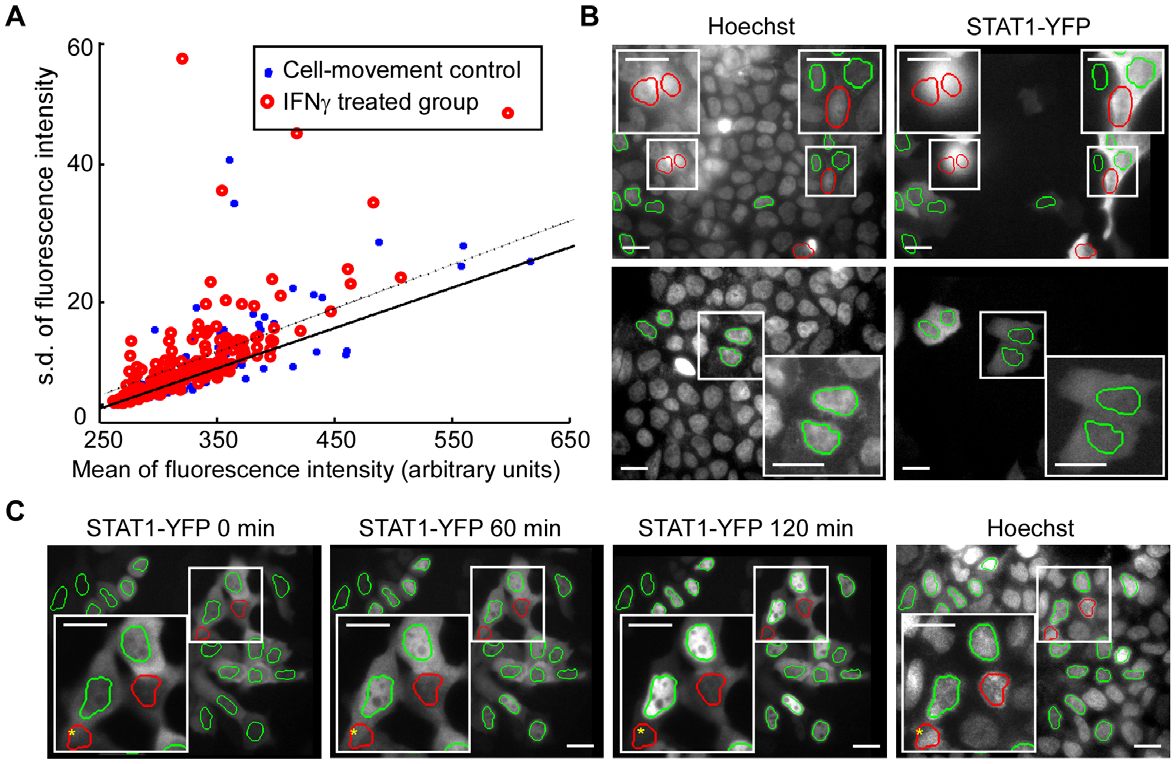
Identification of non-motile cells by classification of the nuclear masks. (A) The s.d. was plotted against the mean for each of the measured YFP fluorescence in the cell-movement control, and the first images of the IFNγ treatment group. The data pairs from the cell-movement control were fitted to a solid line: y = 0.07821×x -16.83 with y s.d. and x mean. The dotted line represents the 99.5% confidence interval (CI) of the fit. The figure shows a typical result from three independent experiments, from 310 and 324 nuclei in the cell-movement control and treatment groups, respectively. (B) Examples of good segmentation (upper panel) and bad segmentation (lower panel) from typical images from the cell-movement control. (C) Representative segmentation effects in the time-lapse images at t = 0, or at 60 and 120 min after IFNγ treatment. The masks which generated data pairs from processing of the first image (0 min) that are within the CI are outlined in green, ones outside the CI are in red. For each image, magnified views of selected cells are shown in insets. Stars denote false masks. Arrows indicate identified mismatched masks at time 0. Images with black borders are registered images; the borders indicate the distance by which the original YFP images are shifted away from the fixed-cell images. Scale bar, 20 µm.

Based on these data, we designed an algorithm to identify immobile cells by classifying nuclear masks. In this attempt to investigate rules of the nuclear fluorescence of inactivate STAT1-YFP, 800 nuclear masks were randomly selected from the cell-movement control in three independent experiments. The s.d. and mean of the measured nuclear fluorescence were plotted (Fig. 4A, closed blue circles), and the relationship was accessed by the robust fitting linear regression [25]. This technique uses an iteratively reweighted least-squares algorithm and is less sensitive to outliers than ordinary linear regression [25], [26]. We identified a relationship between the mean and the s.d. by a fit, y = 0.07821×x -16.83 with y s.d. and x mean (Sum of squared error: 286.5; R-square: 0.9498; Adjusted R-square: 0.9494; root mean squared error: 1.364) (Fig. 4A, solid line). As mentioned above, the results from the artificially mismatched masks indicated that the s.d. increased more significantly than the mean (Fig. 3B, squares). Two different skilled professionals were employed to manually associate the relationship with the segmentation effects. The s.d. and mean resulting from 93.92% matched masks were found to fall inside the 99.5% CI of the fit, while the data pairs from over 80.00% mismatched and false segmented masks fell outside (Fig. 4A, closed blue circles; Fig. 4B; Table S1). Therefore, the upper boundary of the fit was applicable to categorize the nuclear masks into two groups: matched, and mismatched or false masks. In the future, it might be feasible to use an upper boundary of a selected CI of the fit to categorise the masks into the two groups more conveniently.

### Application of the method: identification of cells with morphological changes

We next applied the CI to the time-lapse images. Because the image segmentation was based on the nuclear-staining of the fixed cells, the nuclear masks were likely to decrease the degree of match with the corresponding nuclei from the end point to the first image in a time-lapse image sequence. Therefore, the data pairs from the first image should best represent the segmentation effect in the whole image sequence, and these data were thus classified by the CI into matched and mismatched masks (Fig. 4A, red circles). The two different skilled professionals were then employed to evaluate the classification effect by judging the results of 800 nuclear masks, randomly selected from three independent time-lapse experiments. The results showed that 88.75% were matched masks, 90.00% of which fell inside the CI. Mismatch and false segmentation accounted for 11.25%, and over 84.00% of this condition fell outside the CI. A comparison indicated that 58 (7.25%) and 30 (3.75%) masks were mismatched in the time-lapse and cell-movement control groups, respectively (Tables S1 and S2). This suggested that about 3∼4% cells changed shapes or moved obviously from their original positions during the time lapse. Typical images are illustrated in Fig. 4C. These data demonstrate that the method identifies moving cells accurately (how cell motility and false segmentation could bias the final results will be discussed subsequently). In addition, the results demonstrate that few cells are motile after IFNγ treatment.

### Optimizing the method: comparison of descriptors for quantifying STAT1 nuclear translocation

**Figure 5:**
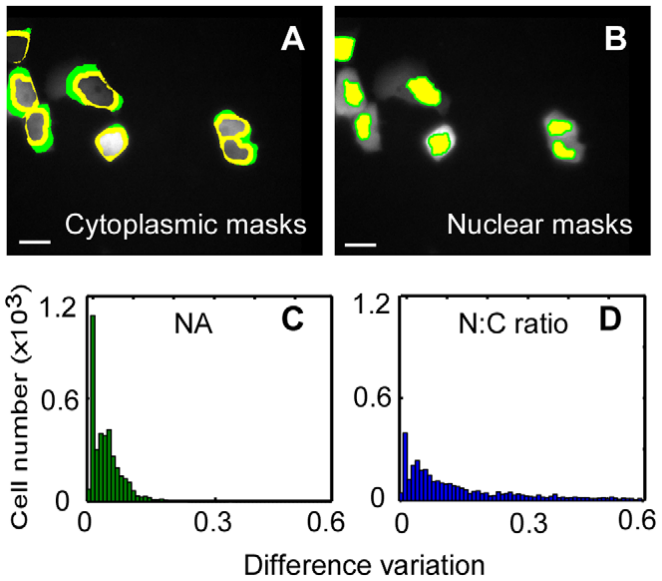
Comparison between NA and N∶C ratio for measuring STAT1 nuclear translocation. To evaluate the utility of these two parameters in describing STAT1-YFP nuclear import in time-lapse images of IFNγ treated Hela cells, two sets of masks, cytoplasmic and nuclear, were produced by regulating the erosion and dilation (Image analysis). Here shows a typical STAT1-YFP image obtained at 120 min after IFNγ administration and its masks. One set of the mask was labeled in yellow, the other in both yellow and green (A, B). With the two sets of the masks, NA was employed to quantify STAT1 nuclear translocation, generating two data sets. The distribution of the difference variation between the two data sets is shown as a histogram (C). Likewise, the distribution of the difference variation of N∶C ratio was calculated (D). Results are from 32 sets of time-lapse images with 430 cells from a representative experiment. Images with black borders are the registered images; the borders indicate the distance by which the original YFP image is shifted away from the fixed-cell images. Scale bar: 20 µm.

To identify the most accurate parameter with which to track nuclear translocation of STAT1, a conventional descriptor, N∶C ratio [6], [7], [27], [28], was compared with NA (detailed in **Material and Methods**). We first produced two sets of masks (Fig. 5A and 5B) and obtained two data sets (two values for each cell in each image) to analyze STAT1 nuclear translocation using NA. Then the difference variation between the two data sets was calculated. Likewise, the difference variation representing N∶C ratio was obtained. The difference variation resulting from NA (0.0524; median) was found significantly smaller than that from N∶C ratio (0.1015; median) (Wilcoxon signed-rank test, paired, two-sided, *p* = 0.0001; data against the normal distribution tested by the Jarque–Bera test, *p* = 0.0010). Similar results were obtained by repeating six times with different masks. A typical result is shown in Figure 5. We thus used the more reliable descriptor, NA, to quantify the nuclear import of STAT1.

As far as image segmentation and biological characteristics are concerned, there are a number of reasons why NA is preferable descriptor to N∶C ratio in time-lapse imaging. For segmentation, cell cytoplasm is much more difficult to contour than the nucleus [12], [29], so introducing cytoplasmic segmentation certainly brings in more errors. From the biological point of view, cytoplasmic fluorescence varies more significantly among cells, autofluorescence is more intense in cytoplasm (data not shown), and, with regard to live-cell images, the cytoplasmic shape changes more extensively than the nuclear shape. However, N∶C ratio is more informative in describing fluorescence distribution between the nucleus and cytoplasm in an individual cell, and was therefore still used in our study for this purpose.

### Patterns of STAT1-YFP nuclear import

**Figure 6:**
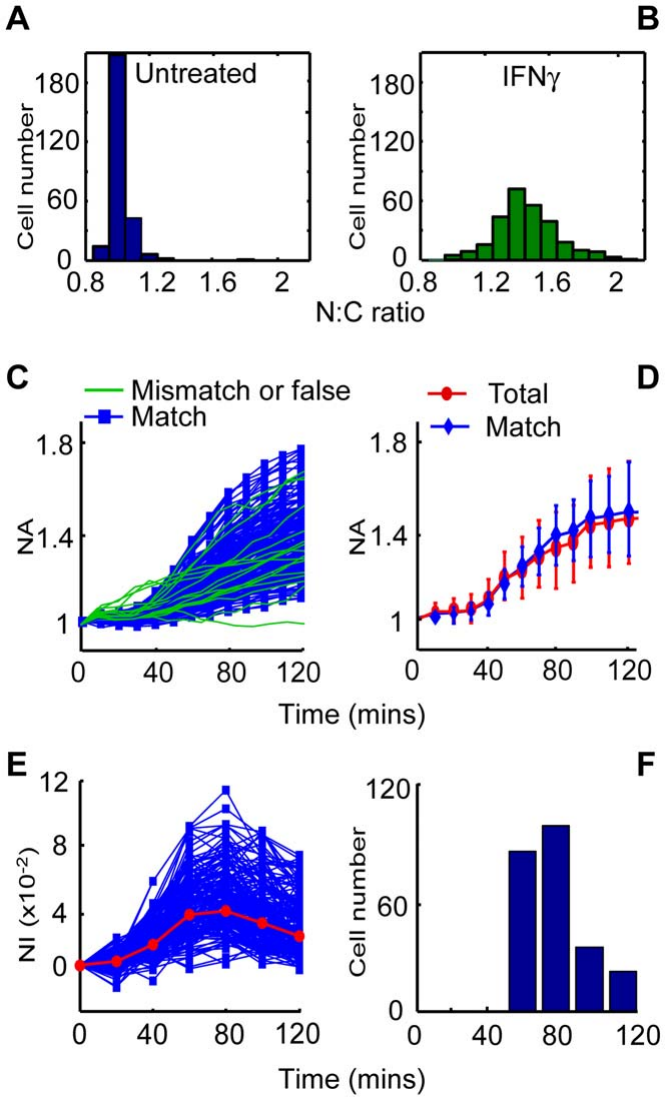
Time course of STAT1-YFP import into nuclei. Hela cells transiently expressing STAT1-YFP were treated with IFNγ for 120 min. Before treatment, STAT1-YFP fluorescence was mainly detected in the cytoplasm, the distribution of the N∶C ratios is shown as a histogram (**A**), the distribution shifted to **B** after the treatment. (**C**) For each cell that was responded to IFNγ (see “**Patterns of STAT1-YFP nuclear import**” for detail), the nuclear accumulation (NA) was quantified at the indicated time in the time course. Blue represents cell nuclei that were well segmented (match), and green represents cell nuclei that were motile or falsely segmented (mismatch or false). (**D**) Shows descriptive statistics (mean and standard deviation) of the data from **C**. Red represents all cells identified (total), including cells that were well segmented, falsely segmented and motile cells. Blue represents stationary cells in the time course (match). (**E**) The time course of STAT1-YFP nuclear import (as indicated by **C**, blue lines) was measured by nuclear increment (NI). The curves in red represent the median values in the time intervals showing the tendency. (**F**) Time required for STAT1-YFP to achieve maximum speed of translocation, and each bar represents the time point at which NI reached the maximum speed in the indicated number of cells. NI is more sensitive to noise than NA, so a 20-minute interval was used. Shown is a typical result from three independent experiments with 360 cells.

STAT1-YFP functions the same as endogenous STAT1 as far as nuclear translocation is concerned [17], [30]. Figure 6A shows the distribution of the N∶C ratio before IFNγ stimulation, which does not conform to the normal distribution (the Jarque–Bera test, *p* = 0.0000). Therefore the central tendency was measured by the median (0.9965), and the difference between the 5th and 95th percentile of the distribution was employed to indicate the extension (0.8781, 1.2110). Maximum nuclear translocation of STAT1-YFP was seen about 2 hours after IFNγ addition (Fig. 4C), and the N∶C ratios exhibited a central tendency (1.2914) and extension (1.1226, 2.1956) (Fig. 6B). Because less than 5% cells had N∶C ratio greater than 1.2110 (the 95th percentile) before the treatment, the cells with higher ratio after 120 min were deemed to have responded to IFNγ.

Parallel time-lapse images of untreated cells for fluorescence variation did not exhibit any significant fluorescence variation in this process (data not shown), therefore the change in fluorescence can reflect the movement of STAT1 in response to IFNγ. Due to the non-homogeneity of STAT1-YFP import, only cells which responded to IFNγ were included to model STAT1 nuclear translocation, in order to minimize noise caused by the unresponsive cells. The increase of the nuclear fluorescence was found to vary significantly in magnitude (NA, Fig. 6C, blue lines) and speed (NI, Fig. 6E) from cell to cell, but exhibit a common feature of three-stage movement as shown in Figure 6D (blue lines) and 6E. During the first 20 minutes, nuclear translocation was barely detectable, suggesting a lag period. This was followed by an acceleration stage lasting from about 20 to 70∼100 minutes, which included a slow start and then a jump to the maximum speed(Fig. 6E and 6F; each bar represents the time point at which NI reached the maximum speed in the indicated number of cells). Finally, a decelerating stage occurred, manifested by a plateau of NA (Fig. 6D, blue lines) and a decrease of NI (Fig. 6E).

The significance of removing motile and falsely segmented cells on the final readout was also evaluated. For doing this, we treated motile and falsely segmented cells together, because our algorithm identified these two conditions as a whole. The dynamics of STAT1 nuclear accumulation showed that the cells which were motile or falsely segmented tended to have higher N∶C ratios at the beginning of the nuclear import but lower ratios at the end (Fig. 6C). Statistics also suggested that removing these biases resulted in a smoother curve of nuclear accumulation against time (Fig. 6D). Another impact of these conditions on the readout involved higher extension, as revealed by s.d. in Figure 6D. As can be expected, the bias generated by cell motility and false segmentation was trivial (Fig. 6C and 6D), because these problem cells only accounted for a small proportion of the cell population (less than 12% as mentioned above). However, we think removing motile cells should be increasingly important in cases that more cells are motile in experiments which require a long time-lapse or use cells that move more efficiently.

This three-stage movement of STAT1 import is in agreement with knowledge of how IFNγ signals to STAT1. The IFNγ receptor consists of two subunits, interferon-γ receptor 1 (IFNΓR1) chain and interferon-γ receptor 2 (IFNΓR2) chain. The binding of IFNγ to IFNΓR1 leads to formation of IFNΓR1/IFNΓR2 complexes, resulting in transphosphorylation and activation of intracellular JAK kinases. Phosphorylation of IFNΓR1 generates a docking site for STAT1, which is then phosphorylated by the JAK kinases. Phosphorylated STAT1 dissociates from the protein complex, homodimerizes, and migrates into the nucleus [31]. The time lapse between IFNγ addition and STAT1 migration may represent the process of IFNγ binding with IFNΓR1, the formation of the IFNΓR1/IFNΓR2 complexes, and the activation of JAK kinases. The following accelerating phase might indicate the time period for the maximum activation of the JAK kinases. Finally, the decrease of cytoplasmic STAT1 molecules and/or possibly the gradual deactivation of the JAK kinases would leads to reduction of STAT1 nuclear import.

### Factors for heterogeneities of cell responses to IFNγ

**Figure 7:**
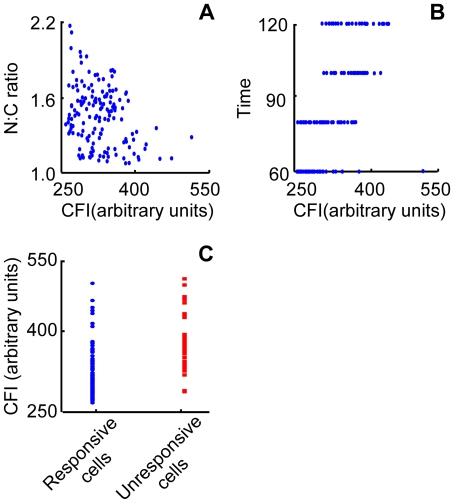
Correlation between cell heterogeneity to IFNγ and initial STAT1-YFP expression level. Time-lapse images of Hela cells expressing STAT1-YFP were captured before and after 2 hours of IFNγ administration. (**A**) Nuclear∶cytoplasmic ratio (N∶C ratio) of STAT1-YFP fluorescence at 2 hours after the treatment was plotted against the cytoplasmic fluorescence intensity of the same cells before the treatment. (**B**) Relationship between the times required for STAT1-YFP to reach maximum nuclear translocation speed and the fluorescence intensity before stimulation. (**C**) STAT1-YFP fluorescence level in responsive and unresponsive cells. Cytoplasmic fluorescence intensity, CFI. Shown here is a typical result with 394 cells from three independent experiments.

The heterogeneous responses of the cells might be associated with several cell phenotypes, including expression level of STAT1-YFP, or nuclear area, or cell cycle stage, which can be indicated by the fluorescence intensity of Hoechst dye [32]. For verification, the Spearman rank correlation was applied to show whether any of these factors correlated with N∶C ratio at the end of the time-lapse. Only the initial cytoplasmic expression level of STAT1-YFP was found to correlate negatively with the degree of the translocation (two-tailed, *r* = −0.2162; *p* = 0.0000) (Fig. 7A), and correlate positively with the time required to reach the maximum speed (two-tailed, *r* = 0.3121; *p* = 0.0027) (Fig. 7B). We then considered whether the cells with higher STAT1-YFP expression were resistant to IFNγ. To test this, the STAT1-YFP expressing cells were divided into two groups - responsive and unresponsive (in the section of Patterns of STAT1-YFP nuclear import). The fluorescence intensities in the responsive group were found to be significant lower than those in the unresponsive group (Wilcoxon rank sum test, two-sided, *p* = 0.0031) (Fig. 7C). The result suggests that, when STAT1-YFP was expressed at higher levels, the nuclear import took more time to reach the maximum speed, the nuclear accumulation decreases, and cells tended to be unresponsive to IFNγ. The reasons behind the heterogeneous responses of the cells might be related to the kinetics of JAK kinase activity – increase of substrate (STAT1-YFP) leads to longer reaction (phosphorylation) time. This is in agreement with one of our findings regarding the negative correlation between initial STAT1-YFP expression level and nuclear accumulation. In the future, studies with the readout of JAK kinases could link more closely the responses of STAT1, JAKs, and the nuclear accumulation of STAT1.

This study aimed at establishing an automated quantification method for nuclear translocation by time-lapse imaging; a benefit of the quantification is that we were able to document precisely STAT1 heterogeneity in response to IFNγ. Investigating the biological basis of the heterogeneity was not a goal of our study. Nonetheless, our study suggests unrecognized regulations in the signal pathway due to the following findings. 1) Higher expression prolonged the time required for STAT1-YFP nuclear import to reach a maximum speed; 2) Cells with highest STAT1-YFP expression responded slowly to IFNγ. In setting up the method, we also observed that HeLa cells only underwent minor morphological changes hours after IFNγ treatment. The method was thus applicable to investigate nuclear translocation of STAT1-YFP in live HeLa cells, and proved to be reliable for kinetic analysis.

### Conclusion

To the best of our knowledge, this retrospective method provided for the first time an automated solution to quantify nuclear translocation of molecules in living cells in a large scale. Our method is featured by high convincing segmentation based on retrospective nuclear staining of fixed cells, an optimized parameter to measure cytoplasmic-nuclear translocation, and the ability to identify motile cells. This approach works in contexts where cells undergo minor morphological changes during an experiment. Besides HeLa cells, a wide range of cell lines, including Chinese hamster ovary (CHO) cells, 16-human bronchial epithelium (16-HBE) [33], and Human lung adenocarcinoma epithelial (A549) cells are all promising candidates for our automated time-lapse imaging analysis system. STAT1 is just one example of many nuclear-cytoplasmic shuttling proteins that are mostly located in the cytoplasm. Other examples include Smads [34], NF-kappaB p65 subunit [35], glucocorticoid receptor [36], and Signal transducers and activators of transcription 3 (STAT3) [30]. Therefore, the automated solution is broadly applicable to study the dynamic of nuclear translocation of a wide range of shuttle proteins in living cells. Overall, the method has significant advantages in being objective, automated, and very simple, hence can be expected to open the field of time-lapse imaging of nuclear translocation for comprehensive studies. (Readers may consider two questions. 1) The use of Hoechst 33342 to stain live cells may be an easier method to provide nuclear segmentation for the time-lapse images; 2). Because observing a decline of STAT1-YFP fluorescence after fixation (Fig. 2), low levels of a protein might be invisible after fixation. In this instance, this retrospective method might fail in the step of the image registration. We discuss these two questions in the Text S1).

## Supporting Information

Table S1 **Classification of 800 segmented nuclei randomly selected from the cell-movement control in three independent experiments.**
(DOC)

Table S2 **Classification of 800 segmented nuclei randomly selected from the treatment group in three independent experiments.**
(DOC)

Text S1 **This file include: 1) options for shuttle proteins that express at very low level, 2) reasons why Hoechst staining in living cells are not preferred.**
(DOC)

File S1 **The compressed file is the software (Matlab codes) package of the image registration.** There are the Matlab codes, examples (full set of time-lapse and fixed-cell images which are shown in Figure 4) and usages of the image registration. The users can test it and validate it.
(7Z) Click here for additional data file.
