# Supplementary material for: Automated Analysis of Time-Lapse Imaging of Nuclear Translocation by Retrospective Strategy and Its Application to STAT1 in HeLa Cells
Source: PLoS One. 2011 Nov 18;6(11):e27454. doi: 10.1371/journal.pone.0027454 (PMC3220678; doi:10.1371/journal.pone.0027454)
Supplement: Table S1 — Classification of 800 segmented nuclei randomly selected from the cell-movement control in three independent experiments. (DOC) [file pone.0027454.s001.doc]

**Table S1. Classification of 800 segmented nuclei randomly selected from the cell-movement control in three independent experiments**

| Classification | Cell population | Cells inside 99.5%CI | Cells outside 99.5%CI | Percentage of correct classification |
| --- | --- | --- | --- | --- |
| Match segmentation | 740 | 695 | 45 | 93.92% |
| Mismatch segmentation | 30 | 4 | 26 | 86.67% |
| False segmentation | 30 | 6 | 24 | 80.00% |

CI indicates confidence interval of the linear fit: y = 0.07821 × x -16.83 with y s.d. and x mean.
