# Supplementary material for: Automated Analysis of Time-Lapse Imaging of Nuclear Translocation by Retrospective Strategy and Its Application to STAT1 in HeLa Cells
Source: PLoS One. 2011 Nov 18;6(11):e27454. doi: 10.1371/journal.pone.0027454 (PMC3220678; doi:10.1371/journal.pone.0027454)
Supplement: Table S2 — Classification of 800 segmented nuclei randomly selected from the treatment group in three independent experiments. (DOC) [file pone.0027454.s002.doc]

**Table S2. Classification of 800 segmented nuclei randomly selected from the treatment group in three independent experiments**

| Classification | Cell population | Cells inside 99.5%CI | Cells outside 99.5%CI | Percentage of correct classification |
| --- | --- | --- | --- | --- |
| Match segmentation | 710 | 648 | 72 | 90.00% |
| Mismatch segmentation | 58 | 8 | 50 | 86.21% |
| False segmentation | 32 | 5 | 27 | 84.38% |

CI indicates confidence interval of the linear fit: y = 0.07821 × x -16.83 with y s.d. and x mean.
