# Supplementary material for: Automated Analysis of Time-Lapse Imaging of Nuclear Translocation by Retrospective Strategy and Its Application to STAT1 in HeLa Cells
Source: PLoS One. 2011 Nov 18;6(11):e27454. doi: 10.1371/journal.pone.0027454 (PMC3220678; doi:10.1371/journal.pone.0027454)
Supplement: Text S1 — This file include: 1) options for shuttle proteins that express at very low level, 2) reasons why Hoechst staining in living cells are not preferred. (DOC) [file pone.0027454.s003.doc]

**Option for shuttle proteins exhibiting faint fluorescence**

A decline of STAT1-YFP fluorescence was observed after fixation (Fig. 2), so a target protein might be invisible if it exhibited faint fluorescence before fixation. In this instance, this retrospective method could fail in the step of the image registration (Fig. 1B, image registration between Ifixed_YFP and In_YFP). This issue is expected to be solved by one more step. For doing this, Hoechst 33342 can be used to stain the live cells at the end of an experiment, images of the Hoechst staining (Iextra_Hoechst) and an extra YFP (Iextra_YFP) were then taken. Afterwards the cells were fixed and stained by Hoechst. In this case, the reference image to register Ifixed_Hoechst should be the living staining of Hoechst 33342 (Ilive_Hoechst). While the extra YFP image (Iextra_YFP) would be the reference to register In_YFP (Fig. 1B).

Although Hoechst 33342 enable to stain live cells, we still prefer segmentation using Hoechst staining in fixed cells. One drawback of using live staining is the variability of dye uptake in different cells, which may decrease image contrast. Indeed, we found that images generated by Hoechst staining fixed-cells exhibited more uniform signals and higher contrast compared with live-cell staining by Hoechst (data not shown). Moreover, treated cells might be fragile, rapid procedures that maintain cells as close as possible to their natural state should provide the most reliable results. Hoechst has toxic effect on cells [1], and to stain live-cell by Hoechst, incubation time was documented ranging from 10 to 30 minutes [2,3,4]. For our cells, we found at least a 30-minute incubation with Hoechst was required to obtain stable live-cell staining (data not shown). Instead it only took less than 3 minutes from the end of the time-lapse experiment to start the fixation (Material and Methods; Fixed-cell imaging). Therefore, if live-cell staining by Hoechst was performed, cells should undergo more morphological changes. The detection was proved by our observation (data not shown).

**References**

1. Durand RE, Olive PL (1982) Cytotoxicity, Mutagenicity and DNA damage by Hoechst 33342. J Histochem Cytochem 30: 111-116.

2. Martin RM, Leonhardt H, Cardoso MC (2005) DNA labeling in living cells. Cytometry A 67: 45-52.

3. Scatena M, Almeida M, Chaisson ML, Fausto N, Nicosia RF, et al. (1998) NF-kappaB mediates alphavbeta3 integrin-induced endothelial cell survival. J Cell Biol 141: 1083-1093.

4. Purschke M, Rubio N, Held KD, Redmond RW (2010) Phototoxicity of Hoechst 33342 in time-lapse fluorescence microscopy. Photochem Photobiol Sci 9: 1634-1639.
